# Lack of Effects of Toll-Like Receptor 4 Antagonists on the Reinforcing Effects of Cocaine and Remifentanil

**DOI:** 10.4172/2329-6488

**Published:** 2016-12

**Authors:** Takato Hiranita

**Affiliations:** Division of Neurotoxicology, National Center for Toxicological Research (NCTR), U.S. Food and Drug Administration (FDA), AR 72079-9501, USA

## Editorial

The toll like receptor 4 (TLR4) is expressed in glial cells and reacts to potential toxic entities. This activation triggers various inflammatory reactions. (+)-Naloxone and (+)-naltrexone, dextrorotatory enantiomers of opioid receptor antagonists [respectively, (−)-naloxone and (−)-naltrexone], have been demonstrated to dock to TLR4 using “*in-silico* ” models, and function as an antagonist at this site *in vivo* [1,2]. Recently, (+)-naloxone have been reported to antagonize self-administration of a μ-opioid agonist remifentanil [3]. Further, (+)-naltrexone blocked self-administration of a dopamine uptake inhibitor cocaine [4]. These findings suggested a “novel” TLR4-mediated mechanism underlying the reinforcing effects of drugs of abuse across pharmacological classes. A more recent study [5] has, however, indicated that the TLR4 hypothesis is less viable.

Tanda and his colleagues further assessed specificity of the blocking effects of the TLR4 antagonists on self-administration of remifentanil or cocaine [5]. Consistent with the results from the two earlier studies [3,4], both (+)-naloxone and (+)-naltrexone dose-dependently produced an insurmountable antagonism against self-administration of remifentanil or cocaine. However, the antagonism was accompanied with a dose-dependent decrease in food-reinforced responding. For example, (+)-naloxone and (+)-naltrexone were equipotent in decreasing responding maintained by injections of remifentanil or cocaine or presentations of food pellets. Thus, the apparent antagonism of the TLR4 antagonists against drug self-administration is likely due to a non-specific disruption of overall behavioral performance rather than an interaction with the reinforcing effects of cocaine or remifentanil. On the other hand, cocaine was not reinforcing under a fixed- and progressive-ratio schedule of reinforcement in TLR4 mutant mice whereas sucrose was reinforcing [4]. Thus, TLR4 signaling appears to mediate somehow cocaine reinforcement.

The more recent results by Tanda et al. [5] indicate the following message: 1) There is no specific preclinical efficacy of TLR4 antagonists (+)-naloxone and (+)-naltrexone for self-administration of cocaine and remifentanil. Thus, the TLR4 hypothesis is less viable to develop medications for drug abuse; 2) It is important to assess pharmacological specificity using food-reinforced responding as successfully employed in previous studies [5–9].
